# Possible risks posed by single‐stranded DNA viruses of pigs associated with xenotransplantation

**DOI:** 10.1111/xen.12453

**Published:** 2018-08-18

**Authors:** Anbu K. Karuppannan, Tanja Opriessnig

**Affiliations:** ^1^ Department of Veterinary Diagnostic and Production Animal Medicine College of Veterinary Medicine Iowa State University Ames Iowa; ^2^ The Roslin Institute and The Royal (Dick) School of Veterinary Studies University of Edinburgh Roslin Midlothian UK

**Keywords:** cross‐species, porcine circovirus (PCV), porcine parvovirus (PPV), torque teno sus virus (TTSuV), xenotransplantation, zoonotic

## Abstract

Routine large‐scale xenotransplantation from pigs to humans is getting closer to clinical reality owing to several state‐of‐the‐art technologies, especially the ability to rapidly engineer genetically defined pigs. However, using pig organs in humans poses risks including unwanted cross‐species transfer of viruses and adaption of these pig viruses to the human organ recipient. Recent developments in the field of virology, including the advent of metagenomic techniques to characterize entire viromes, have led to the identification of a plethora of viruses in many niches. Single‐stranded DNA (ssDNA) viruses are the largest group prevalent in virome studies in mammals. Specifically, the ssDNA viral genomes are characterized by a high rate of nucleotide substitution, which confers a proclivity to adapt to new hosts and cross‐species barriers. Pig‐associated ssDNA viruses include torque teno sus viruses (TTSuV) in the *Anelloviridae* family, porcine parvoviruses (PPV), and porcine bocaviruses (PBoV) both in the family of *Parvoviridae*, and porcine circoviruses (PCV) in the *Circoviridae* family, some of which have been confirmed to be pathogenic to pigs. The risks of these viruses for the human recipient during xenotransplantation procedures are relatively unknown. Based on the scant knowledge available on the prevalence, predilection, and pathogenicity of pig‐associated ssDNA viruses, careful screening and monitoring are required. In the case of positive identification, risk assessments and strategies to eliminate these viruses in xenotransplantation pig stock may be needed.

## 1 INTRODUCTION

The first successful xenograft implantation of a chemically treated pig heart valve into a human was carried out more than 50 years ago, and pigs are now a major source of these bioprosthetics.^1^, ^2^ Similarly, xenotransplantation of porcine Langerhans islet cells to diabetic humans was first attempted in 1994, and efforts to make this an effective therapy are ongoing.^3^, ^4^ Owing to these precedents, pigs are considered the preferred donor species for xenotransplantation to humans, with recent promising trials of successful porcine kidney and heart xenotransplantation to nonhuman primates.^5^, ^6^ Particularly, pigs are inexpensive, easy to breed in a controlled environment with large litter sizes, and the organ size of pigs is comparable to that of humans. However, immunological rejection of xenograft from pigs has been a major issue in the past.^6^, ^7^

Developments in genome editing technology, such as the wide availability of the CRISPR‐Cas9 system, have opened avenues to engineer the pig genome and create immunologically safe organ donors for humans in the near future.^8^ Apart from the compatibility and immunological rejection of the xenotransplant, microbial safety is also a primary concern in xenotransplantation. Pigs are a relatively distant species to humans compared to nonhuman primates, and overall, there is a perceived lower risk of cross‐species pathogen transmission. However, pigs are known to be a reservoir of viruses that are pathogenic to humans such as Japanese encephalitis virus, Nipah virus, swine influenza A virus, Menangle virus, and Hepatitis E virus.^9^, ^10^, ^11^, ^12^, ^13^, ^14^ In addition to these viruses, porcine cytomegalovirus and vesicular stomatitis virus are also known to have zoonotic potential.^15^, ^16^

Cross‐species transmissions of viruses between pigs and humans can be broadly described to occur under three scenarios. The first “classical scenario” is facilitated by shared ecosystems of humans and pigs and a certain degree of susceptibility of humans to these viruses.^9^, ^12^ This scenario includes exposure of humans to pig viruses via the food chain, farming, and veterinary activities. In the second scenario, humans are exposed to pig viruses without being in the proximity of pigs, through products of pig origin used in pharmaceutical products for human patients. These products may include anticoagulants, respiratory agents, and digestive supplements that contain or are based on pork by‐products. In addition, human vaccines may be prepared on porcine cell lines or use porcine‐derived cell culture supplements. Administration of these can lead to unintended exposure of humans with pig virus contaminants such as the well‐documented case of porcine circovirus 1 (PCV1) in live‐attenuated rotavirus vaccines.^17^

However, xenotransplantation poses an altogether different third scenario where tissues or organs from pigs are placed directly inside the body of the human recipient, who is likely under immunosuppressive treatment. Viruses that are being passaged with the xenotransplant may continue to replicate in the xenografted organ and could replicate in the host as well. Therefore, the risk of exposure to viruses from donor pigs to the xenotransplant recipient is rather unique. Such viral contaminants may result in active infections of host tissues and can contribute to problems in the engraftment of the transplanted tissue such as inflammation and adverse immune reactions.

## 2 PIG SELECTION FOR XENOTRANSPLANTATION

There is a wide availability of diagnostic reagents and well‐established monitoring programs for selected pathogens important to commercial pig production. In contrast, pigs bred and maintained for xenotransplantation under high biosecurity procedures require a different approach to ensure inadvertent transmission of porcine viruses to the recipient. A real concern in xenotransplanation is the presence of asymptomatic viral swine infections, which are not part of routine pig veterinary screening. Viruses of pigs which are of potential risk in xenotransplantation have been widely researched and reviewed.^15^, ^16^ Some of these viruses cause overt infections, others such as porcine lymphotropic herpesvirus 1 (PLHV‐1), PLHV‐2, and PLHV‐3 may cause latent infections thereby complicating any screening processes. Yet other viruses such as porcine endogenous retrovirus C (PERV‐C) are integrated in the genome of some pigs, which makes screening for them easy.^16^ PERV‐A and PERV‐B, integrated in the genome of all pigs and human‐tropic, may infrequently produce infective particles and can infect human cells as well as cells of other species. On the other hand, the pig‐tropic and infective PERV‐C, which is not found in all pigs, can recombine with chromosome integrated PERV‐A and produce infective viruses.^18^ PERV‐A/C recombinants are also human‐tropic, but their replication rate is much higher compared to the parental strains.^18^, ^19^ They are absent in the germline, but integrate de novo in cells of different organs.^19^, ^20^ Differences in the chromosomal copy number of PERV in different organs in an individual pig indicate that these viruses are still active, replicate to produce infectious particles, and can infect and integrate de novo at other sites.^18^, ^20^ For PERV specifically, advances in genome engineering techniques have led to the generation of cell lines in which all genomic PERV material has been disrupted, and these cells have been used to produce transgenic pigs by somatic cell nuclear transfer.^8^, ^21^ This comprises a major achievement in overcoming the previous obstacle of genomic integrated PERVs in xenotransplantation. However, re‐infection of such PERV disrupted pigs with PERV‐C or recombinant PERV‐A/C remains a risk. In the case of other infectious viruses with no obvious effect on pig health or production parameters, a mixed strategy for screening could be used. Protocols for genome detection by PCR and antibody detection by serological assays of pig populations and individual pigs intended for transplantation should be devised to prevent cross‐species virus transmission during transplantation.

## 3 SINGLE‐STRANDED DNA VIRUSES IN PIGS

In this review, ssDNA genome viruses in the context of xenotransplantation are being discussed. Under the Baltimore virus classification system, ssDNA viruses are classified as Group II viruses.^22^, ^23^ ssDNA viruses are not enveloped, very resistant to inactivation, can be found in a variety of vertebrate and invertebrate hosts, and have a high mutation rate approaching that of RNA viruses.^22^ The three major families of ssDNA viruses of potential importance in xenotransplantation using pig‐derived organs are *Anelloviridae*,* Circoviridae,* and *Parvoviridae* (Table 1).

**Table 1:** A list of ssDNA viruses prevalent in pigs

Family	Genus	Species	Nucleotide substitutions per site per year	Typical prevalence	Pathogenicity in pigs	Vaccine in pigs	Detection in human cell lines or clinical samples
*Anelloviriade*	*Iotatorquevirus*	*TTSuV 1a*	10^−4^ a	60%^126^	Inconclusive	No	Human PBMCs^49^, ^50^
		*TTSuV 1b*			Inconclusive	No	
	*Kappatorquevirus*	TTSuV k2a		77%^126^	Inconclusive	No	No information
		TTSuV k2b			Inconclusive	No	No information
*Parvoviridae*	*Ungulate protoparvovirus 1*	*PPV1*	10^−4^	67%^72^	Pathogenic	Available	Does not infect cell lines or exposed humans^74^, ^76^
	*Ungulate tetraparvovirus 2*	PPV3	Unknown	39%^72^	Inconclusive	No	No information
	*Ungulate tetraparvovirus 3*	*PPV2*	Unknown	58%^72^	Inconclusive	No	No information
	*Ungulate copiparvovirus 2*	*PPV4*	Unknown	33%^72^	Inconclusive	No	No information
		*PPV5*	Unknown	3%^71^	Inconclusive	No	No information
		*PPV6*	Unknown	6.1%^73^	Inconclusive	No	No information
	*Ungulate bocaparvovirus 2*	*PBoV1*	Unknown	29%^127^	Inconclusive	No	No information
		*PBoV2*	Unknown	7%^127^	Inconclusive	No	No information
		*PBoV6*	Unknown	16%^128^	Inconclusive	No	No information
	*Ungulate bocaparvovirus 3*	*PBoV5*	Unknown	16%^128^	Inconclusive	No	Acute respiratory tract infection in a child^129^
	*Ungulate bocaparvovirus 4*	*PBoV7*	Unknown	55%^72^	Inconclusive	No	No information
	*Ungulate bocaparvovirus 5*	*PBoV3*	Unknown	9%^127^	Inconclusive	No	No information
		*PBoV4‐1*	Unknown	22%^127^	Inconclusive	No	No information
		*PBoV4‐2*	Unknown	31%^127^	Inconclusive	No	No information
*Circoviridae*	*Circovirus*	*PCV1*	10^−5^	2.4%^94^	Not pathogenic	No	HEK 293 cells, HeLa cells, Chang liver cells, Huh‐7 cells^102^, ^104^Exposed children did not develop antibodies^103^
		*PCV2*	10^−3^	82%^94^	Pathogenic	Available	Rd cells^102^
		*PCV3*	Unknown	34.4%^92^	Inconclusive	No	No information

TTSuV, Torque teno sus virus; PPV, porcine parvovirus; PBoV, porcine bocavirus; PCV, porcine circovirus; PBMC, peripheral blood monocytes.

a Estimate from *Anelloviridae* infecting plants.

### 3.1 Anellovirus

#### 3.1.1 Epidemiology in humans and pigs

The members of the *Anelloviridae* have closed circular genomes and typically encode four genes, including a replicase gene which is essential for virus replication.^24^ Anelloviruses are estimated to have a mutation rate of 10^‐4^ nucleotide substitutions per site per year.^25^ The first member of this family, torque teno virus (TTV), was isolated in a human case of post‐transfusion hepatitis.^26^ This virus is now known to be ubiquitous in the human population worldwide, and in one instance has been identified in 94% of analyzed healthy individuals.^27^ Five major phylogenetic clusters of TTV differing mainly in the ORF1 (replicase gene) are observed.^28^ Although the TTV has not been definitively associated with any disease, it is known to suppress the host interferon response.^28^ TTV can replicate to high levels, with an increase in TTV levels being observed in conditions associated with immunosuppression such as human immunodeficiency virus (HIV) infection, untreated solid cancers, and stem cell or solid organ transplantation.^28^, ^29^ The TTV level is inversely correlated with CD8^+^57^+^ T lymphocytes and is thought to be indicative of the status of immunocompetence in humans.^28^, ^30^ A high TTV level is correlated with nonrejection of transplants in allografts.^28^, ^31^ After the discovery of TTV, other members of the *Anelloviridae* such as Torque Teno mini virus (2000) and Torque Teno midi virus (2007) have been identified in human and nonhuman primate populations.^28^, ^32^, ^33^ It is now estimated that *Anelloviridae* constitute around 68% of the virome in healthy humans, making this virus group the largest component.^28^

The Torque teno sus virus (TTSuV) was first discovered in 2002 from healthy pig serum in Japan.^34^ It is now known that TTSuVs are prevalent worldwide and two distinct genera, TTSuV1 and TTSuV2 with 40‐50% sequence identity, have been identified (Table 1). TTSuV1 and TTSuV2 are both further subclassified into two genotypes.^35^, ^36^, ^37^ Antigenic cross‐reactivity is observed between the two genotypes TTSuV1a and TTSuV1b but not between the two species TTSuV1a/b and TTSuV2.^35^ Although varying levels of prevalence of TTSuVs in farmed pigs are reported, prevalence rates generally increase with age and may reach up to 100% in finisher and breeding pigs.^37^, ^38^, ^39^ In addition, natural mixed infections of TTSuV1 and TTSuV2 have been observed.^38^ TTSuVs are transmitted by the oral‐fecal and vertical routes, with the former considered the main route of transmission.^38^, ^40^ TTSuV is also found in boar semen used for artificial insemination, but the importance of this virus transmission route is unknown.^41^

#### 3.1.2 Infection in pigs and tropism

Experimental infections of TTSuV1 and TTSuV2 of gnotobiotic pigs suggest a pathogenic potential of these viruses; however, conclusive evidence of the pathogenicity of TTSuVs in pigs is not established to date.^36^, ^42^ Although a positive correlation between the TTSuV DNA levels and the major pig pathogen porcine circovirus type 2 (PCV2) has been observed in clinically affected pigs, this is interpreted to be an effect of immunosuppression caused by high levels of PCV2.^43^ TTSuV1 and TTSuV2 are distributed in many organs of naturally infected pigs, with highest virus concentration in bone marrow. Moreover, T lymphocytes seem to carry a high TTSuV genome load.^43^, ^44^, ^45^, ^46^ In farmed pigs, organs of potential value in transplantation such as kidney or liver are found to harbor TTSuV1 and TTSuV2 with a prevalence of more than 50%.^43^, ^45^, ^46^ Islet cells in the pancreas could contain TTSuV acquired through circulation; however, this has not been examined. In addition, TTSuV DNA was identified in porcine biologicals such as *Mycoplasma hyopneumoniae* bacterins, porcine products such as trypsin, and in cell cultures derived from different species.^47^, ^48^

#### 3.1.3 Zoonotic potential of TTSuV

Interestingly, TTV DNA is found in pigs and TTSuV DNA is found in humans.^49^, ^50^ Preliminary evidence indicates TTSuV1 replication in human peripheral blood monocytes (PBMCs) and the presence of antibodies against TTSuV1 ORF2 (a nonstructural protein) in human serum samples suggests a zoonotic potential.^50^ Of note, TTSuV1‐infected human PBMCs are shown to be impaired in their mitogenic response.^50^ In addition to humans, TTSuV1 DNA and antibodies against TTSuV1 ORF2 antibodies are found in many mammalian hosts such as horses, cattle, sheep, and dogs, indicative of promiscuity in the host range of TTSuV1.^49^ At present, cell culture propagation of TTSuV is not established. The potential of this virus to infect human‐origin cell lines needs to be further explored, as this may offer clues to its zoonotic potential. Although the pathogenicity of neither TTV nor TTSuVs has been clearly established, the potential of TTSuV to cross the species barrier and suppress the mitogenic response of human PBMCs raises a concern in xenotransplantation.^49^, ^50^

#### 3.1.4 Negative pig sources

Infections with TTSuV are thought to be widely prevalent and persistent; however, fetuses can be negative for TTSuV and it appears feasible to derive TTSuV1 and TTSuV2 negative herds by a combination of cesarean delivery and high biosecurity conditions of rearing.^41^ However, previous contamination of the pig housing facility with Anelloviruses may be an issue and needs to be resolved if existing facilities are being re‐utilized rather than using new buildings for donor pig housing. Currently, there is no commercial vaccine available against TTSuV.

### 3.2 Parvovirus

#### 3.2.1 Epidemiology in humans and pigs

The *Parvoviridae* family consists of eight genera which are characterized by a linear ssDNA genome, ranging from 4 to 6 kb in size, typically with two open reading frames and terminal repeats essential for replication.^51^ Parvoviruses infect a range of vertebrate hosts such as humans, pigs, dogs, cats, and avian species. Viruses in this group have a high mutation rate, especially in the capsid gene, to the order of approximately 10^−4^ nucleotide substitutions per site per year.^52^, ^53^, ^54^, ^55^ This high mutation rate is thought to favor their rapid adaptation to new host species. Parvoviruses are known to have a predilection for rapidly dividing cells, such as bone marrow stem cells and enterocytes.^51^ In particular, the synthesis (S) phase of the cell cycle is thought to benefit their replication.^51^ Human parvovirus B19 (B19V), human bocavirus 1‐4 (HBoV1‐4), and human parvovirus 4 (PARV4) are some of the human viruses in the *Parvoviridae* family considered as pathogenic or a potential risk.^56^, ^57^, ^58^ B19V is associated with acute and chronic infections in healthy and immunocompromised individuals causing disease conditions such as erythema infectiosum, aplastic anemia, and arthropathy.^58^ Although there is no definitive evidence, HBoV is thought to be associated, at least as a coinfection, in respiratory tract infections in children and gastrointestinal tract infections in all age groups.^59^ Similarly, PARV4 is associated with HIV and hepatitis C virus (HCV) infections and is found in higher prevalence in intravenous narcotics users, but a causative link with disease has not been identified.^58^ Human parvoviruses are known to be intransigent to in vitro culture and only a few have been propagated in cell culture such as the B19V and HBoV.^60^, ^61^, ^62^, ^63^ In pigs, eight species of the *Parvoviridae* family classified under four genera are described (Table 1); ungulate protoparvovirus 1 [classical porcine parvovirus (PPV) or PPV1], ungulate tetraparvovirus 2 (PPV3), ungulate tetraparvovirus 3 (PPV2, also known as porcine hokovirus or porcine partetravirus or porcine PARV4), ungulate copiparvovirus 2 (PPV4, PPV5 and PPV6), ungulate bocaparvovirus 2 [porcine bocavirus (PBoV) 1, 2 and 6], ungulate bocaparvovirus 3 (PBoV 5), ungulate bocaparvovirus 4 (PBoV 7), and ungulate bocaparvovirus 5 (PBoV 3, PBoV4.1 and PBoV4.2).^64^, ^65^ PPV1 was first identified in the early 1970s in association with abortions in pigs.^66^, ^67^, ^68^ However, it has also been associated with enteric disease and skin infections in growing pigs.^69^ In pigs, PPVs and PBoVs, in line with the corresponding viruses infecting mammals, have a high rate of mutation.^70^

#### 3.2.2 Infection in pigs and tropism

Other than for PPV1, pathogenicity of any of the newly identified PPVs or PBoVs has not yet been conclusively demonstrated. Parvovirus DNA can be detected, even in healthy pigs, in a wide range of pig tissues including tissues of possible interest for xenotransplantation such as liver and kidney.^71^ The PPVs and PBoVs are considered possible triggers for the development of systemic disease in PCV2‐infected pigs.^72^ A survey in the United States using random samples submitted for diagnostics from 2006 to 2013 showed the prevalence of PPV in tissues as follows: 15.2% for PPV1, 42.7% for PPV2, 9.1% for PPV3, 4.3% for PPV4, and 3.0% for PPV5.^71^ In another study conducted in Japan, PPVs were detected in high frequencies as coinfections in PCV2‐infected pigs; PPV1 coinfection was found in 67% of the pigs, PPV2 in 58%, PPV3 in 39%, PPV4 in 33%, and PBoV7 was detected in 55% of the pigs.^72^ Overall, the studies show that PPV1 and PPV2 are the most prevalent parvoviruses in pigs.^71^, ^72^, ^73^ PPV1, PBoV3, and PBoV4 are the only pig‐associated *Parvoviridae* members that have been cultured in vitro.^74^, ^75^

#### 3.2.3 Zoonotic potential of PPV

Porcine parvovirus 1 does not infect cell lines of human or primate origin.^74^ Hemophilia patients treated with porcine clotting factor VIII, in which PPV1 DNA was detected, did not develop any antibodies to PPV1.^76^ There is no information on the ability of other PPVs to replicate in human cells. However, the general ability of parvoviruses to cross‐species barrier has been well noted among virologists.^77^, ^78^ The history of emergence of canine parvovirus (CPV) from feline panleukopenia virus (FPV) and its further evolution into novel genotypes with varying host range (CPV2a, b and c) is a paradigm in virology.^77^, ^79^ The evolution of CPV has been recapitulated in vitro and a single mutation is sufficient to allow replication in a novel host cell.^77^ Similarly, the rodent H1 parvovirus is well studied for its oncolytic potential in human cancer cells.^80^, ^81^ The ability of the H1 virus to selectively infect human cancer cells is thought to be determined by the capsid protein and enabled by the increased rate of proliferation of the cancerous cells and their subdued antiviral mechanisms.^81^, ^82^ In the case of oncolytic rodent H1 parvovirus, its ability to replicate in human cells is proof of having sufficient factors for the virus to replicate and produce an infective progeny H1 virus in cancerous cells. Given the rapid mutation rate of PPVs and the overall ability of parvoviruses to cross‐species barriers, the risk of widely prevalent PPVs in a xenotransplantation setting, where the recipient will undergo prolonged immunosuppressive regimen, is worth closer scrutiny.

#### 3.2.4 Negative pig sources

While vaccines are available for PPV1 and widely used in breeding herds, they do not prevent infection but rather protect against disease.^83^ As the PPVs and PBoVs are not found in all sows and piglets, derivation of free piglets is feasible and the chances can be improved by cesarean section and colostrum deprivation. However, these viruses are widespread and very resistant to disinfection; thus, contamination of the pig housing facility may be difficult to resolve and could result in infection of piglets early in life.

### 3.3 Porcine circovirus

#### 3.3.1 Epidemiology in humans and pigs

Porcine circoviruses (PCV) are small, icosahedral, nonenveloped virus particles with a circular ssDNA genome of 1.7‐2 kb size. The genomes of members of the *Circoviridae* family are characterized by a stem‐loop structure at the origin of replication and encode for replicase proteins and a single capsid protein in opposite orientations in addition to other putative genes.^24^ These are referred to as circular rep‐encoding ssDNA (CRESS‐DNA) genomes.^24^ The replicase protein consists of endonuclease and helicase motifs and is essential for the replication of the circoviral genome by rolling circle replication.^22^ Circoviruses have the highest rate of mutation among ssDNA viruses, and the PCV2 genome is estimated to incur 1.2 × 10^−3^ nucleotide substitutions per site per year.^22^, ^84^ Although no specific circoviruses of humans have been reported, cycloviruses and gemycircularviruses, classified under the *Cyclovirus* genus of the *Circoviridae* family, have been detected in cerebrospinal fluids of humans.^85^ In pigs, three different species have been identified, PCV1, PCV2, and PCV3. PCV1 was discovered as a contaminant of the porcine kidney cell line PK‐15 in 1974 and is today considered nonpathogenic to pigs.^86^, ^87^ In the late 1990s, PCV2 was identified as a major pig pathogen associated with a series of disease outbreaks in North America and Europe.^88^, ^89^, ^90^ PCV2 is an essential etiological agent that, along with other coinfecting pig pathogens, causes immunosuppression that leads to postweaning multisystemic wasting syndrome (PMWS).^91^ PCV3 is a more recently identified member of the genus identified across the world without any conclusive link to pathogenicity.^92^, ^93^ In a U.S. National Animal Health Monitoring System's (NAHMS) pig study conducted in 2006, a total of 6234 serum samples were collected from farms with 100 or more pigs and these sera were analyzed for PCV1 and PCV2 DNA.^94^ While over 82% of sera from 185 farms were positive for PCV2 by PCR, only 2.4% were positive for PCV1. More than 80% of PCV2 DNA‐positive pigs were also positive for anti‐PCV2 antibodies.^94^ Comprehensive data on prevalence of the novel PCV3 are yet being generated. However, similar to PCV1 and PCV2, it is considered to be prevalent worldwide.^92^

#### 3.3.2 Infection in pigs and tropism

Under experimental conditions, PCV1‐infected pigs remain clinically healthy and do not develop histopathological lesions. However, PCV1 antigen is detected in multiple organs and tissues and PCV1 viremia can be detected in sera for up to 35 days postinoculation.^95^, ^96^, ^97^ Experimental infection of pigs with PCV2 alone is similar to PCV1 infection and is almost always subclinical. However, in combination with other factors including infectious agents, PCV2 infection can lead to manifestation of clinical disease in a percentage of infected pigs, with typical microscopic lesions of mild‐to‐sever histiocytic‐to‐granulomatous inflammation of multiple organs, formation of multinucleated giant cells, and lymphoid depletion.^96^ Viral tissue load is generally high in clinically affected PCV2‐infected pigs and includes organs of interest for xenotransplantation such as liver, kidneys, and pancreas, while in subclinically infected pigs, PCV2 replication is often limited to individual lymph nodes. Efficacious vaccines against PCV2 that prevent pathogenesis and reduce PCV2 viremia and shedding are available, but as with most vaccines, they do not prevent infection of pigs.^98^

#### 3.3.3 Zoonotic potential of PCV

The threat posed by PCVs during xenotransplantation has been previously reviewed.^99^ PCV1 and PCV2 are both propagated mainly in the porcine kidney cell line PK‐15 but can be also cultured in other cells of porcine origin.^100^, ^101^, ^102^ Under extreme in vitro conditions, it has been reported that PCV1 undergoes nonproductive replication in human cell lines (293, HeLa, and Chang liver cells), in which PCV1 replication and gene expression were detected but infectious virus particles are not produced.^102^ Human blood leukocytes reportedly infected by PCV1‐like particles were visualized by electron microscope and PCV1 DNA was detected in cells; however, infectivity was not determined.^103^ In another study, productive PCV1 infection in a subclone of the human hepatocellular carcinoma cell line (Huh‐7, subclone 10‐3) was observed.^104^ With regard to PCV2, experimental infection was observed in human Rd cells.^102^

Porcine circovirus DNA has been detected in U.S. human stool samples and approximately 5.3% (13/247) were positive for PCV1 or PCV2 DNA.^105^ This finding is thought to reflect dietary consumption of PCV2‐containing pork products rather than infection.^105^, ^106^ Noninfectious PCV1 has been detected in commercial pepsin, commercial pig vaccines, and in U.S. pork products.^105^, ^107^, ^108^ PCV1 DNA was detected in Madin‐Darby bovine kidney **(**MDBK) cells, which are used to grow selected animal vaccine virus strains.^109^ Thirty‐one of 88 cell lines of various origin (cattle, pig, monkey, hamster, rat, mouse, rabbit, cat, sheep, canine, human, equine, and insect) and one in ten trypsin samples used for cell culture were positive for PCV1 DNA.^110^ In January 2010, an academic research team discovered PCV1 DNA in the oral live‐attenuated human rotavirus vaccine, Rotarix^™^ (GlaxoSmithKline [GSK] Vaccines).^17^ However, there was no immunological or clinical evidence of PCV1 infection in infants who had received Rotarix^™^ in clinical trials.^111^

In summary, PCVs have been widely prevalent in the global swine population for many decades. However, there is no conclusive evidence of human infections with PCV1 or PCV2 despite being constantly exposed to PCV by various routes and even in high‐risk groups such as swine veterinarians.^112^, ^113^

#### 3.3.4 Negative pig sources

Breeding stock free of PCV1 and PCV2 has been derived by a combination of screening for PCV2 DNA, colostrum deprivation, and enhanced biosecurity in husbandry.^112^, ^114^, ^115^ Secondary exposure of PCV2 naïve pigs to environmental contamination of the facility is the greatest challenge when maintaining PCV2 free populations, and it can be very difficult to decontaminate existing facilities.

## 4 DISCUSSION

In general, ssDNA viruses infecting humans and pigs are widely prevalent and have high mutation rates. However, this high prevalence may not hinder the use of pig xenografts, as a recent report highlights that islet cells of pigs do not carry common pig viruses including PCV2 and PPV1, even if other cells such as PBMCs carry them.^116^ Indeed, xenotransplantation of islet cells from pigs to cynomolgus monkeys in preclinical trials and from pigs to human patients in clinical trials has been achieved without transmission of any pig viruses including ssDNA viruses such as PPV, PCV1, and PCV2.^117^, ^118^ However, it should be noted that the donor pigs used in these studies, Auckland Island pigs of mixed European genetic heritage, were from a specific pathogen‐free breeding unit and confirmed PCV1 and PCV2 negative.^117^, ^118^

Although currently, there is no explicit evidence of human infection and pathogenesis associated with pig ssDNA viruses of the *Anelloviridae*,* Parvoviridae* and *Circoviridae* families, precautions to address these viruses in xenotransplantation may need to be considered including careful screening and monitoring. Derivation of ssDNA virus‐free stock is possible by a combination of rigorous screening of breeding stock and piglets, cesarean section to deliver piglets, and colostrum deprivation. Recommendations and guidelines for donor pig‐related testing have been summarized by the International Xenotransplantation Association.^119^ Prevention of infection of the derived virus‐free stock is a more difficult task which requires a high level of biosecurity in housing and management practices.^112^, ^114^ All nonenveloped ssDNA viruses are hardy, survive in the environment for prolonged periods, and are resistant to commonly used disinfectants.^120^, ^121^, ^122^, ^123^ Animal handlers should wear barrier overalls, gloves, and masks to prevent transmission of these ubiquitous viruses from the field to virus‐free stock. In addition to the pig housing facility, equipment, water, feed, and veterinary supplies are potential sources of contamination and should be screened and decontaminated thoroughly.^114^, ^120^

Currently, commercial kits for surveillance of ssDNA viruses by serology and PCR are available only for PPV1 and PCV2. However, primer sequences for detecting other pig ssDNA viruses by quantitative PCR are widely available in the literature (Table 2). In addition, primer‐free metagenomic sequencing, which is getting more affordable by the day, is a powerful technique to screen for the above viruses. Due to lack of in‐depth knowledge on many aspects of ssDNA viruses, it may be beneficial to screen for them at various levels, such as in the source herd, in donor pigs, harvested organs or cells, and in the donor recipients.^124^ It may also be beneficial to build dedicated facilities to rear such pigs. However, building and maintaining high‐level biosecurity pig units is usually associated with a high cost, and with the current state of knowledge perhaps not justifiable. This will need to be further discussed with regulatory agencies. The majority of the ssDNA viruses infecting pigs have not yet been cultured in vitro. Therefore, the development of pig and human cell line repositories, perhaps genetically engineered to remove innate antiviral defenses, would help in understanding the biology and risk posed by these viruses in xenotransplantation. As ssDNA viruses are ubiquitous in pigs, they could be used as “indicators” to assess the level of “viral load” of the pigs intended for xenotransplantation to humans.^125^

**Table 2:** Primers and probes for quantitative detection of genomes of common ssDNA viruses of pigs

Virus	Primers	Reference
*TTSuV 1a*	TTSuV‐F 5′‐CGAATGGCTGAGTTTATGCC	38 Common primer for all TTSuVs and a specific probe for each species
*TTSuV 1b*	TTSuV‐R 5′‐GATAGGCCCCTTGACTCCG	
*TTSuV k2a*	TTSuV1‐Probe‐ 5′‐ AACTGTCTAGCGACTGGGCGGGT‐3′	
*TTSuV k2b*	TTSuV2‐Probe 5′‐AACAGAGCTGAGTGTCTAACCGCCTG‐3′	
*PPV1*	PPV1 F 5′‐CAGAATCAGCAACCTCACCA‐3′ PPV1 R 5′‐GCTGCTGGTGTGTATGGAAG‐3′ PPV1‐Probe 5′‐TGCAAGCTTAATGGTCGCACTAGACA‐3′	130
*PPV2*	PPV2‐DF 5′‐TACTGAGCCCTAAGACTGACTACAAGC‐3′ PPV2‐DR 5′‐GTTTGTCTCGTTGTTCGTCTGATG‐3′ PPV2‐Prob5′‐AACTGCTACATGAACCA CTTTACCCCSTC‐3′	131
*PPV3*	PPV3F 5′‐CAYGAYGAACGGTACGATGAAAT‐3′ PPV3R‐ 5′‐GCGGTAAAACCTGTGAWAWTTGAAC‐3′ PPV3 Probe 5′‐TAGGTTGATGAATAAGGAGATAGAGAGGGCGG‐3′	132
*PPV4*	PPV4&5 F 5′‐GCATTGGTGTGTGTCTGTGTCC ‐3′ PPV4&5 R 5′‐GTGGCACATTTGTACATGGGAG‐3′	133 Common primers for PPV4 and PPV5 and a specific probe for each species
*PPV5*	PPV4 probe 5′‐ CTCCGCGGGATGTGCTTACAATTTTCA ‐3′ PPV5 probe 5′‐ ACTTTGGTGTTGAGGGACTTAGCTTTTTTGTAC ‐3′	
*PPV6*	PPV6F5′‐GGCTTCATAATCCCTCCAAAACCT‐3′ PPV6R5′‐GCTCATCTTCCTCTTGTTTCTCCTG‐3′ PPV6probe 5′‐CCTCCTCCTCCTCCCTCTCCAATTCCT‐3′	73
*PBoV G1*	G1F5′‐TGAGCTAATCCCTGAACTG ‐3′	134 Primers for use in quantitative real‐time PCR with DNA intercalating fluorescent dyes such as SYBR Green or EVA Green
	G1R5′‐GTCTGAGCCTGTATCACCTAT‐3′	
*PBoV G2*	G2F5′‐GGGCACTGATTATATCTTTAC‐3′	
	G2R5′‐CCCTGACATCTTTCCATT‐3′	
*PBoV G3*	G3F5′‐ACTCTTTGCAGTCTGACTCT′TC‐3′	
	G3R‐ 5′‐GTTCCCCCGTGTCTTTAG‐3′	
*PCV1*	PCV1 F 5′‐TGG CCC GCA GTA TTT TGA TT ‐3′ PCV1 R5′‐CAG CTG GGA CAG CAG TTG AG ‐3′ PCV1 Probe5′‐CAG CAA TCA GGC CCC CCA GGA AT ‐3′	135
*PCV2*	PCV2 F‐ 5′‐CAG CTG GGA CAG CAG TTG AG ‐3′ PCV2 R‐5′‐TGG CCC GCA GTA TTT TGA TT ‐3′ Probe 5′‐CCA GCA ATC AGA CCC CGT TGG AAT G ‐3′	136
*PCV3*	PCV3 F‐5′‐AGT GCT CCC CAT TGA ACG‐3′ PCV3 5′‐ACA CAG CCG TTA CTT CAC‐3′ PCV3 probe 5′‐ACC CCA TGG CTC AAC ACA TAT GAC C–3′	93

## CONFLICT OF INTERESTS

The authors declare no conflict of interests.

## AUTHORS’ CONTRIBUTIONS

Anbu K. Karuppannan drafted the manuscript and both Anbu K. Karuppannan and Tanja Opriessnig reviewed the literature and revised the final manuscript.
